# Inflammatory mediators in nasal secretions of patients with nasal polyposis with and without aspirin sensitivity

**DOI:** 10.1002/iid3.791

**Published:** 2023-02-23

**Authors:** Aleksandar Perić, Dejan Gaćeša, Gordana Cvetković, Danilo Vojvodić

**Affiliations:** ^1^ Department of Otorhinolaryngology, Faculty of Medicine of the Military Medical Academy University of Defence Belgrade Serbia; ^2^ Department of Otorhinolaryngology ENT Hospital „Dr. Žutić“ Belgrade Serbia; ^3^ Department of Pulmonology, Faculty of Medicine of the Military Medical Academy University of Defence Belgrade Serbia; ^4^ Division of Clinical and Experimental Immunology, Faculty of Medicine of the Military Medical Academy Institute for Medical Research Belgrade Serbia

**Keywords:** aspirin, eosinophils, mast cells, nasal polyps, respiratory mucosa

## Abstract

**Background:**

The aim of this cross‐sectional study was to compare the levels of inflammatory mediators in nasal secretions in patients with aspirin‐exacerbated respiratory disease (AERD) and in those with nasal polyposis (NP) without aspirin‐sensitivity and to correlate nasal fluid mediator concentrations with clinical parameters of the disease.

**Methods:**

A total of 30 patients with AERD, 30 chronic rhinosinusitis (CRS) with NP patients without aspirin sensitivity (CRSwNP), and 30 control subjects without inflammation of the nasal mucosa (C), selected for surgical treatment entered the study. The total nasal symptom score (TNSS), endoscopic score (ES), and Lund‐Mackay score (LMS), were evaluated. The concentrations of eosinophil cationic protein (ECP), tryptase, heat shock protein 70 (HSP70), substance P and Clara cell protein 16 (CC16) were determined in nasal secretions.

**Results:**

Higher concentrations of ECP, tryptase, and HSP70 were measured in the AERD patients than in the CRSwNP patients and the C group (*p* < .001; *p* < .001, respectively for all mediators). However, levels of CC16 were higher in the C group than in the AERD and CRSwNP groups (*p* < .001; *p* < .001, respectively). A positive correlation between the TNSS and CC16 and a negative one between CC16 and tryptase levels were found in the C group. The CRSwNP group showed positive correlations between ECP, HSP70, and tryptase and negative correlations between substance P, ES, and LMS, as well as between CC16 and tryptase levels. In the AERD group, we found a positive correlation between HSP70 and ECP levels and a negative correlation between the TNSS and CC16 concentration.

**Conclusion:**

The obtained results indicate the increased production of mediators of eosinophil and mast cell function, and the decreased production of biomarker of respiratory epithelial function in AERD patients. Clinical and biochemical parameters correlate in different ways in the AERD and CRSwNP patients.

## Graphical abstract

Our results showed higher concentrations of ECP, HSP70 and tryptase in AERD patients than in nonaspirin sensitized CRSwNP patients and in subjects with healthy nasal mucosa. Clinical and biochemical parameters of chronic inflammation correlate in different ways in AERD and nonaspirin sensitized CRSwNP patients, suggesting the posibility that different inflammatory mechanisms are involved in the development of these two forms of disease.

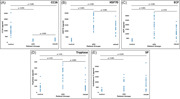

## 1 INTRODUCTION

Nasal polyposis (NP) or chronic rhinosinusitis with nasal polyps (CRSwNP) is an inflammatory disease of the nasal and sinus mucous membranes in which symptoms last longer than 12 weeks.^1^ Although the etiology and pathogenesis have not yet been well understood, the role of bacterial biofilm, innately reduced resistance of the respiratory epithelium, allergy, as well as staphylococcal enterotoxins that locally trigger a strong immune response are under consideration.^1^, ^2^ In addition to hypertrophy of the respiratory epithelium and stromal edema, in more than 90% of European and North American patientsthe disease is histologically characterized by strong infiltration of mucosal tissue with eosinophils, mast cells and other inflammatory cells and immunologically characterized by domination of T2 immune response.^1^, ^2^ Those cells produce inflammatory mediators which induce structural changes in the nasal and sinus mucosa.^1^, ^2^ Among other mediators, eosinophil cationic protein (ECP), which is secreted by eosinophils, tryptase, secreted by mast cells, and heat shock protein 70 (HSP70), released from necrotic and apoptotic cells, play major roles in tissue damage to the nasal mucosa.^1^, ^2^ In addition, HSP70 stimulates the production of a number of proinflammatory mediators, thus maintaining chronic inflammation.^3^, ^4^, ^5^ On the other hand, Clara cell protein 16 (CC16), which is produced by specialized secretory Clara cells, situated within the respiratory epithelium, has a strong anti‐inflammatory effect and participates in the repair process of the nasal mucosa.^1^, ^6^ Neurogenic inflammation of the nasal mucosa is mediated by the release of neuropeptides, especially substance P, at the endings of sensory nerve fibers, and, apart from allergic and nonallergic rhinitis, it also plays a role in the pathogenesis of NP.^7^, ^8^ Within the NP, we distinguish a special clinical phenotype in which CRSwNP is associated with hypersensitivity to nonsteroidal anti‐inflammatory drugs (NSAIDs) and nonallergic asthma. This aspirin‐exacerbated respiratory disease (AERD) is characterized by a particularly severe clinical feature with rapid disease progression and frequent relapses relatively soon after endoscopic surgical treatment.^1^, ^2^, ^9^, ^10^

Many mediators detected in nasal secretions can demonstrate the level of inflammation in the nasal mucosa and progression of chronic disease. By reviewing the literature, we have not found studies to compare the production of proinflammatory and anti‐inflammatory mediators between the two clinical phenotypes of NP, with and without hypersensitivity to NSAIDs, as well as the degree of their association with the clinical parameters of the disease. Considering the more severe clinical course of the disease in AERD patients, we assumed that the production of proinflammatory mediators in them is more intense, while the production of anti‐inflammatory mediators is weaker. In the present study, we compare the levels of ECP, tryptase, HSP70, CC16, and substance P in nasal secretions of the patients with NP with no sensitivity to NSAIDs, the patients with AERD, as well as of the subjects without nasal inflammation. We also investigate a possible correlation between the concentrations of these mediators in nasal secretion, as well as the eventual presence of a correlation between the levels of these mediators and the clinical parameters of NP extent.

## 2 MATERIALS AND METHODS

### 2.1 Ethical consideration

This cross‐sectional investigation was conducted in accordance with the Helsinki Declaration. The methods used in the study were approved by Ethics Committee of our tertiary care institution (IRB Approval No. 21/2022). A written informed consent was obtained from all patients. The study was conducted between March 2018 and October 2022 at the Department of Otorhinolaryngology and Institute for Medical Research of the Military Medical Academy in Belgrade, Serbia. We used the STROBE reporting method to present the results.^11^

### 2.2 Patient selection

This study included patients surgically treated in our hospital during the above‐mentioned period. Patients were selected for the study in the course of an examination in the ENT outpatient clinic, 1 month before admission for surgical treatment. They were diagnosed with CRSwNP in accordance with the European Position Paper on Rhinosinusitis and Nasal Polyps (EPOS) 2020^1^ and the American Academy of Otolaryngology—Head and Neck Surgery (AAO‐HNS)^12^ guidelines. On the same day, pulmonology procedures were performed to assess the status of the lower respiratory tract. The patients were then advised not to take any therapy that may affect the profile of inflammatory mediators in the nasal mucosa/nasal secretions. Final clinical scoring and nasal secretion sampling were performed on the day of admission to the hospital, 1 month after the outpatient clinic procedures. Patients with NP whose bilateral score was 4 and more, were selected for our investigation. All the subjects were examined by a pulmonologist on the day of admission, and the presence/absence of asthma was based on the patient's medical history, clinical data and on pulmonary function testing. Among the patients with polyps, we distinguished a group of patients with AERD. The criteria for the inclusion of AERD patients were: (i) the diagnosis of CRSwNP; (ii) mild persistent asthma, diagnosed by a pulmonologist according to the Global Initiative on Asthma (GINA) guideline^13^; and (iii) medical history information about the worsening of respiratory symptoms after taking one of the NSAIDs. Those with severe persistent asthma were excluded due to the necessity of taking systemic corticosteroid therapy, which affects the concentrations of inflammatory mediators. The second group of patients with NP consisted of patients selected for surgical treatment, in whom asthma and hypersensitivity to NSAIDs were excluded based on medical history, rhinological and pulmonological examinations and pulmonary function tests. The control group consisted of patients selected for surgery due to nasal obstruction caused by pneumatization of the middle turbinate and septal deformation, although they had no symptoms, local and computed tomography (CT) findings of inflammation of the nasal mucosa and paranasal sinuses.

Criteria for exclusion from the study: people younger than 18 and older than 65 years, pregnant women, nursing mothers, patients with systemic diseases affecting the nasal cavity/sinuses (Churg‐Strauss syndrome, granulomatosis with polyangiitis, sarcoidosis, etc.), patients with choanal polyps, hamartomas and fungal rhinosinusitis, patients with a congenital disorder (cystic fibrosis, primary ciliary dyskinesia, etc.), smokers, patients with previous surgery of the nose/sinuses, subjects who took topical and/or systemic corticosteroids, antihistamines and antibiotics within a month before the start of the study.

### 2.3 Symptoms, endoscopic, and CT examination

All the patients, including control subjects assessed and scored their five symptoms (nasal obstruction, hyposmia, itching, rhinorrhea and sneezing) at the day of admission to the hospital from 0 to 3: 0—no symptom, 1—mild, 2—moderate, 3—severe, resulting in the maximum Total Nasal Symptom Score (TNSS) of 15, as described in a previous study.^14^

Final assessment of endoscopic finding was performed at the day of admission to the hospital. It was done by the same rhinologist in all the NP patients in a sitting position, using a rigid endoscope. The scores based on the NP size were calculated according to Lildholdt et al.^15^: 0—no disease; 1—mild disease (polyps not reaching the upper edge of the inferior turbinate); 2—moderate disease (polyps reaching between the upper and the lower edges of the inferior turbinate); 3—severe disease (polyps reaching below the lower edge of the inferior turbinate). The maximum bilateral Endoscopic Score (ES) was 6. The patients with the bilateral score of 4 and more were selected for our investigation.

To evaluate the findings from CT scans, the Lund‐Mackay Score (LMS) was used.^16^ CT scan of the paranasal sinuses was performed a few days before admission to the hospital. The opacifications on the CT scans were graded as 0—no opacification, 1—partial opacification and 2—total opacification of the anterior ethmoid, posterior ethmoid, maxillary, frontal and sphenoid sinus. The opacifications of the ostiomeatal complex were scored as 0—not occluded or 2—occluded. The maximum bilateral LMS was found to be 24.

### 2.4 Sampling of nasal secretions and measurement of biochemical parameters

We used a modified absorption technique for sampling of nasal secretions of all the 60 NP patients and 30 control subjects. We did this in a sitting position of the patients and without the use of topical anesthetic. Cotton wool sticks (10 mm long and 4 mm wide) stood for 5 min in the anterior parts of the middle nasal meati, as previously stated.^17^ After being completely soaked with nasal secretions for 5 min, each piece of cotton wool was immersed in the Eppendorf tube containing 1 mL of transfer medium (phosphate‐buffered saline with gentamycin 50 µg/mL, penicillin G 340 U/mL and fungizone 500 µg/mL) (Institute of Virology and Vaccines, Belgrade, Serbia). The samples were thus delivered to the researcher who measured the mediator concentrations, noting that he was not aware of the clinical status of the patients. Thirty minutes later, which was estimated to be necessary for the diffusion of inflammatory mediators to the transfer medium, the samples were centrifuged to separate and precipitate cell elements from the supernatants. The supernatants were frozen at −70°C, for no longer than 2 months, until mediators were detected. Levels of inflammatory mediators were measured in all samples using commercial human ELISA kits (for ECP, Aviscera Bioscience, Inc; for tryptase, Abbexa Ltd.; for HSP70, My BioSource, Inc.; for CC16, Elabscience Biotechnology, Ltd.; for substance P, Ray Biotech). The concentrations of mediators were expressed in picograms/millilitres (pg/mL). The sensitivities of detection, assay ranges and coefficients of variation for inflammatory mediators are presented in Table 1.

**Table 1:** Sensitivities of detection, assay ranges, and coefficients of variation for measured inflammatory mediators.

Mediator	Sensitivity of detection (pg/mL)	Assay range (pg/mL)	Coefficient of variation (%)
ECP	50	56–10,000	4%–6%
Tryptase	3.1	7.8–500	<10%
HSP70	10	125–4000	<15%
CC16	37.5	62.5–4000	<10%
Substance P	100	100–1000,000	<10%

Abbreviations: CC16, Clara cell protein 16; ECP, eosinophil cationic protein; HSP70, Heat shock protein 70.

### 2.5 Strength of the study and sample size calculation

The strength of the study needed to be at least 80% (0.8), and the probability of error of the first type (α) 0.05. Based on the data from the literature,^18^ ECP level is expected to be highly significantly elevated in the patients with NP as compared to the control subjects (320.35 ± 178.32 ng/mL vs. 97.08 ± 42.53 ng/mL; *p* < .001). According to the same paper, as well, we assumed that the standard deviations (*SD*) values would be relatively high. A moderate effect size (0.34) was chosen to calculate the group size. Approximately 90 of the participants (30 in each group) were required to reach statistical significance at the *p* < .05 level between groups. We used the analysis of variance test (analysis of Variance, fixed effects, omnibus, one‐way) with a commercial software (GPower 3.1.).

### 2.6 Statistical analysis

Kolmogorov–Smirnov and Shapiro–Wilk tests were used for the assessment of the data normality. For the assessment of the differences in the clinical and inflammatory parameters between investigated groups, however, we used the Kruskal–Wallis *H* test, which was followed by post hoc Mann–Whitney *U* test. A Spearman's rank correlation coefficient was used for the calculation of statistical relations between those parameters. A *p* value equal or less than .05 was considered statistically significant. The results in figures and Table 2 are represented as medians with 25th and 75th percentiles.

**Table 2:** Numerical data of demographic, clinical, and inflammatory parameters.

Parameters	Controls	CRSwNP	AERD
Participants (*n*)	30	30	30
Male/female ratio	16/14	15/15	17/13
Age (years)*	45.65 (29.00–63.00)	46.75 (29.00–64.00)	45.40 (27.00–62.00)
Nasal symptom score*	5.00 (4.00–7.00)	11.00 (9.00–13.00)^a^	12.00 (9.00–14.00)^a^
Endoscopic score*	0.00	5.00 (4.00–6.00)	6.00 (4.00–6.00)
Lund‐Mackay score*	0.00	16.00 (12.00–21.00)	20.00 (16.00–23.00)
HSP70 (pg/mL)*	206.75 (186.65–275.45)	1210.25 (1125.40–1435.30)^a^	1810.50 (1345.50–2400.70)^a^, ^b^
CC16 (pg/mL)*	910.25 (820.20–1225.35)	255.00 (214.50–310.20)^a^	125.80 (108.20–220.20)^a^, ^b^
ECP (pg/mL)*	225.45 (186.75–302.25)	1123.40 (905.55–1477.75)^a^	1725.70 (1054.10–2384.45)^a^, ^c^
Tryptase (pg/mL)*	31.25 (20.85–73.55)	72.10 (47.60–96.00)^d^	293.20 (225.00–365.25)^b^, ^d^
Substance P (pg/mL)*	185.70 (147.55–208.65)	776.20 (298.40–1175.25)^a^	578.75 (253.35–1138.45)^a^

*Note*: *The results are represented as medians with 25th and 75th percentiles.

Abbreviations: AERD, aspirin‐exacerbated respiratory disease; CC16, Clara cell protein 16; CRSwNP, NP patients with no aspirin sensitivity; ECP, eosinophil cationic protein; HSP70, heat shock protein 70.

^a^ Difference from controls, *p* < .001.

^b^ Difference between NANP and ANP, *p* < .001.

^c^ Difference between NANP and ANP, *p* < .01.

^d^ Differences from controls, *p* < .01.

## 3 RESULTS

This study included a total of 90 patients (60 with NP and 30 control subjects with no inflammation of the nasal mucosa). All our patients underwent surgical treatment. In the group of patients with NP, 30 patients had a form of the disease with (AERD) and 30 patients without sensitivity to NSAIDs (CRSwNP). No significant differences were found in the age and male/female ratio between the investigated groups.

**Figure 1:**
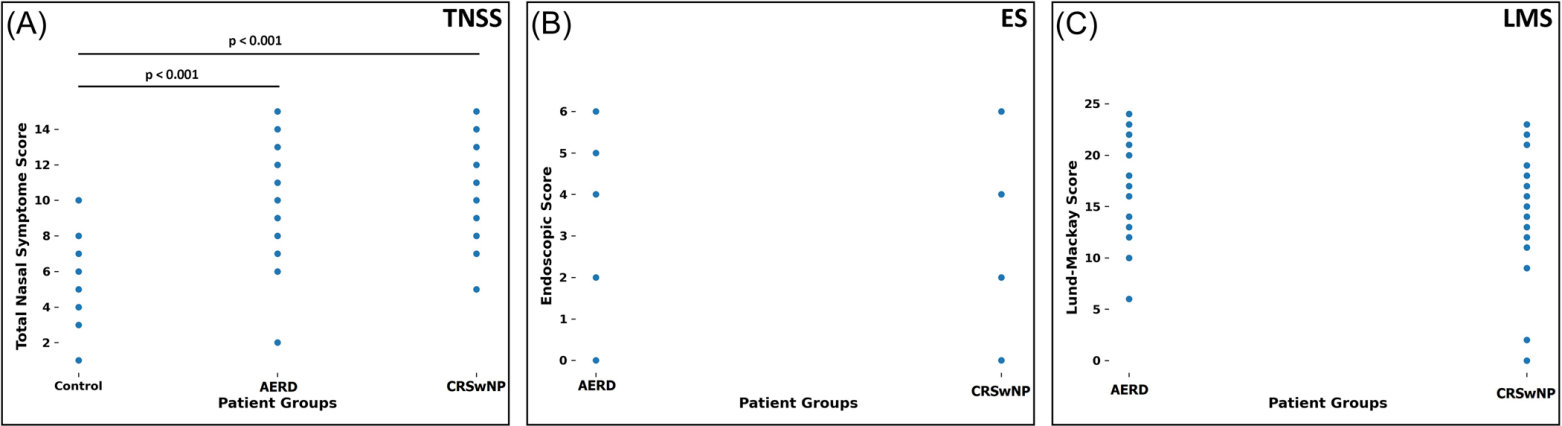
Clinical parameters of patient groups. TNSS was compared between all three groups, while ES and LMS were only assessed in the patients with NP. A statistical difference was shown only for TNSS, as significantly higher both in CRSwNP and AERD groups, when compared with patients from the control group. AERD, aspirin‐exacerbated respiratory disease; CRSwNP, NP without aspirin sensitivity; ES, endoscopic score; LMS, Lund‐Mackay score; TNSS, Total Nasal Symptom Score.

Numerical data of demographic, clinical, and inflammatory parameters are presented in Table 2. By analysis of clinical parameters, we found out a significant statistical difference only for TNSS. A significant difference was found between the groups C and CRSwNP (*p* < .001), as well as between the groups C and AERD (*p* < .001). No difference was found between the groups AERD and CRSwNP (*p* = .597). Likewise, ES and LMS values showed no statistical difference between the groups AERD and CRSwNP (*p* = .134; *p* = .095, respectively) (Figure 1).

**Figure 2:**
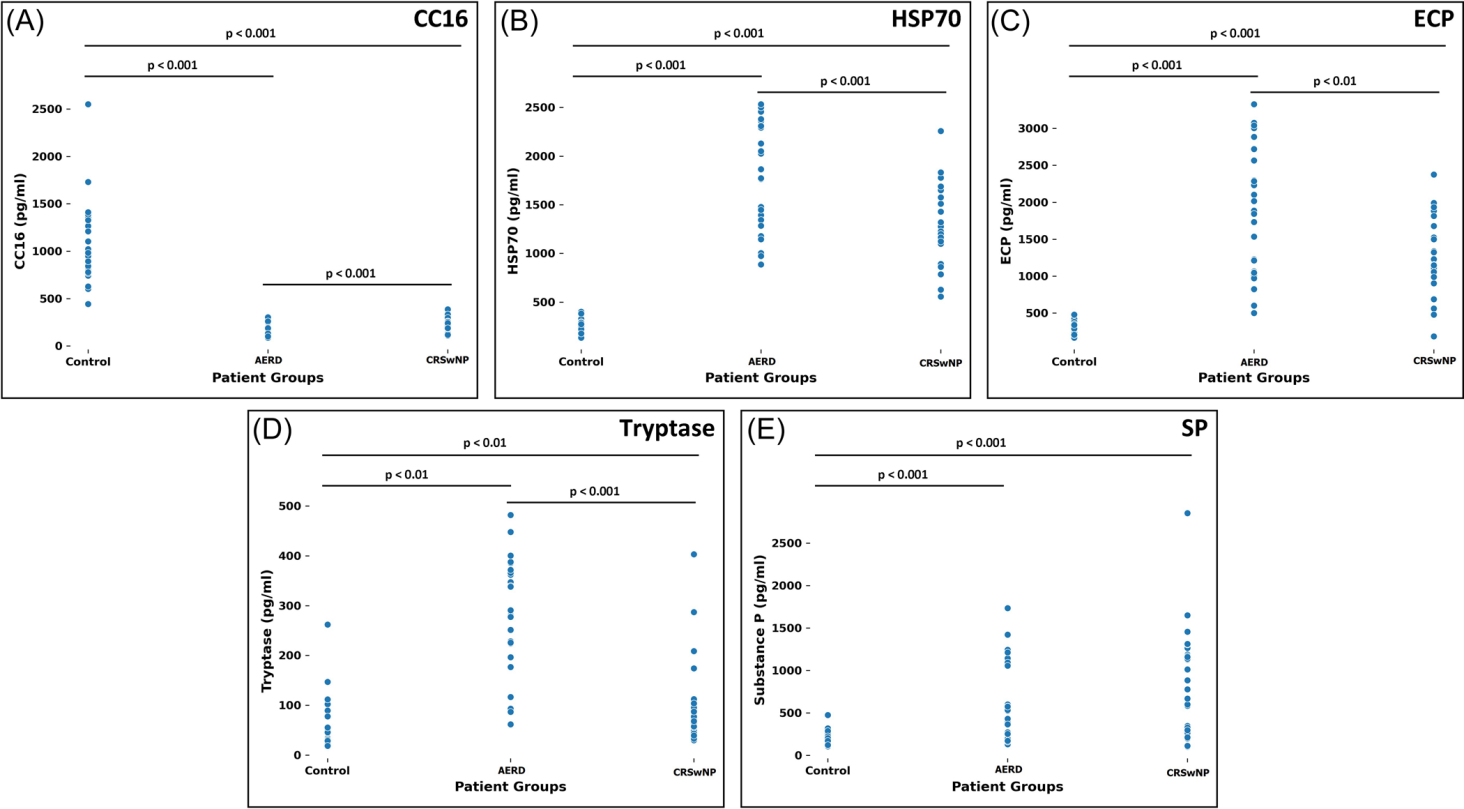
Inflammatory mediators of patient groups. (A) CC16 was significantly higher in control group when compared to CRSwNP and AERD groups. Additionally, CC16 was higher in the CRSwNP than in the AERD group. (B) HSP70 values were significantly lower in the control group when compared to the CRSwNP and AERD groups, while the CRSwNP patients had lower values than the patients from the AERD group. (C) ECP showed lower values in the control group than in the CRSwNP and AERD group and significantly lower values in the CRSwNP group than in the AERD group. (D) Tryptase levels were also lower in the control group than in CRSwNP and AERD groups. This mediator is also lower in CRSwNP than in AERD patients. (E) Significantly lower levels of Substance P were only discovered in the control group, while no significant difference was detected between CRSwNP and AERD groups. AERD, aspirin‐exacerbated respiratory disease; CC16, Clara cell protein 16; CRSwNP, NP with no aspirin sensitivity; HSP70, heat shock protein 70.

The analysis of inflammatory mediator levels showed a significant differences between the groups C and CRSwNP (for CC16: *p* < .001; for HSP70: *p* < .001; for ECP: *p* < .001; for tryptase: *p* < .01; for substance P: *p* < .001), C and AERD groups (for CC16: *p* < .001; for HSP70: *p* < .001; for ECP: *p* < .001; for tryptase: *p* < .001; for substance P: *p* < .001) and between CRSwNP and AERD groups for all mediators except for substance P (for CC16: *p* < .001; for HSP70: *p* < .001; for ECP: *p* < .01; for tryptase: *p* < .001; for substance P: *p* = .34) (Figure 2).

**Figure 3:**
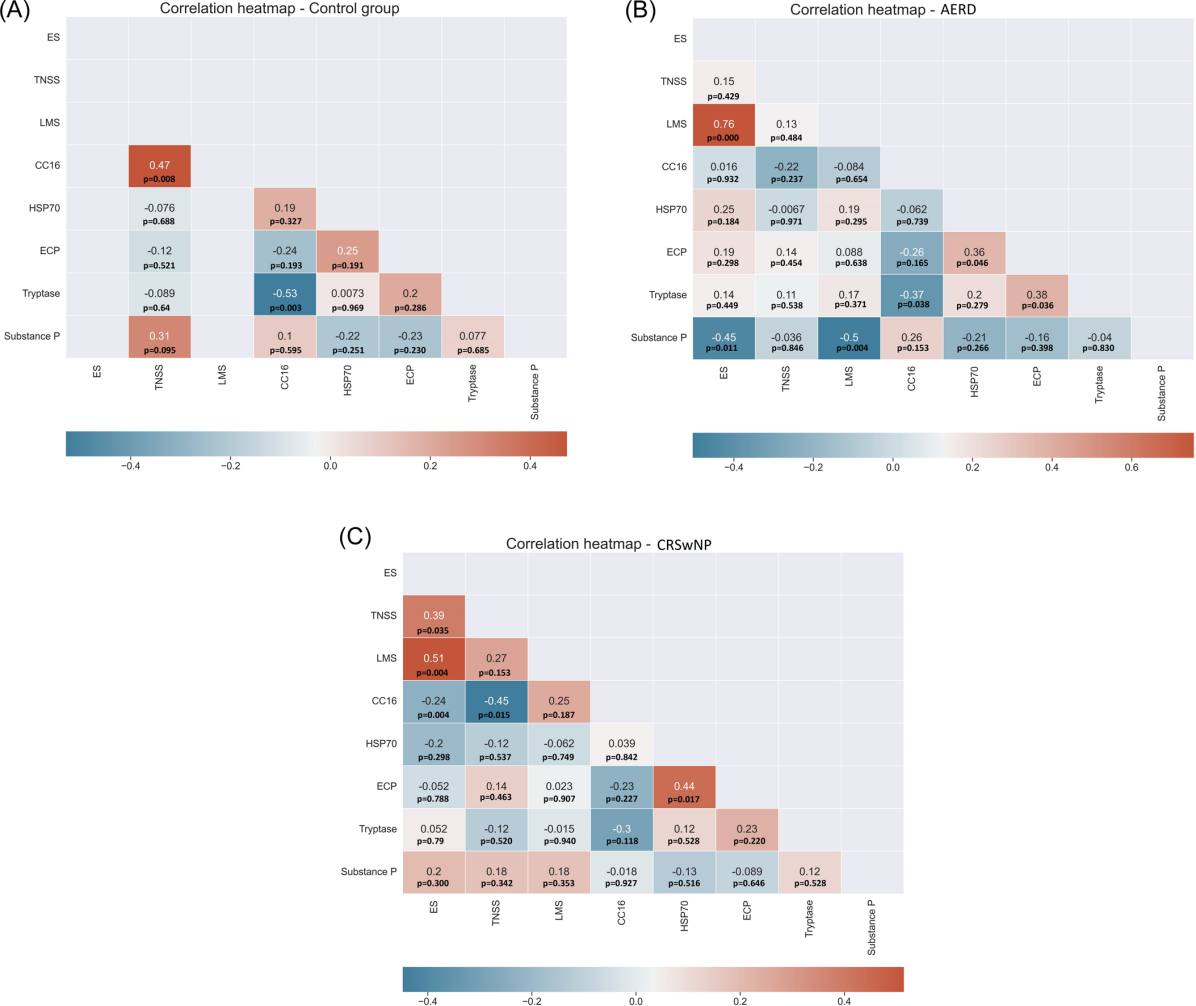
Heatmaps of the correlation between inflammatory mediators and clinical parameters. Colors in the heatmaps correspond to the strength and direction of the correlation, where red color denotes a positive correlation, blue color denotes a negative correlation, while white color denotes a correlation coefficient of 0. Values in the heatmap boxes represent the Spearman's correlation coefficient. (A) Control group; (B) AERD group; (C) CRSwNP group. AERD, aspirin‐exacerbated respiratory disease.

The group C participants showed a positive correlation between CC16 and TNSS and a negative one between CC16 and tryptase in nasal secretions (Figure 3A). In the AERD group, we found positive correlations between TNSS and ES, between ES and LMS, as well as between HSP70 and ECP levels in nasal secretions. On the other hand, a negative correlation was found between TNSS and CC16 levels in nasal fluid (Figure 3B). The patients from the CRSwNP group showed positive correlations between ES and LMS, between HSP70 and ECP, between ECP and tryptase. Negative correlations in the patients from the CRSwNP group were found between substance P and ES, substance P and LMS, as well as between CC16 and tryptase in nasal secretions (Figure 3C).

## 4 DISCUSSION

Chronic inflammation of the nasal mucous membrane is maintained due to the predominance of proinflammatory and reduced activity of anti‐inflammatory mediators. The intensity of eosinophilic activity can be monitored by measuring the concentration of ECP, while the activity of mast cells is best monitored by determining the concentration of tryptase in nasal secretions.^18^, ^19^ Neurogenic inflammation plays an important role in the pathogenesis of allergic and nonallergic chronic rhinitis, although some data suggest a role in the pathogenesis of NP.^7^, ^8^, ^20^, ^21^, ^22^ Monitoring the concentration of substance P in nasal secretions could help us with that. The course of inflammation mediated by proinflammatory mediators in the mucosa of the upper respiratory tract is somewhat limited by the activity of anti‐inflammatory mediators, and one of the most important is CC16.^1^, ^2^, ^6^, ^16^

In patients with AERD, there is a violation of the cyclooxygenase pathway, and an emphasis on the lipoxygenase pathway of arachidonic acid metabolism, which results in the deposition of proinflammatory leukotrienes in the mucosal tissue of the upper and lower respiratory tract.^23^ According to the study by Lyly et al.,^23^ the density of eosinophilic infiltration of polyp tissue increases the risk of revision surgery during the follow‐up of patients with AERD.

The obtained results demonstrate higher concentrations of HSP70, ECP and tryptase in nasal secretions of the patients with AERD than in the NP patients without NSAID‐sensitivity, suggesting the presence of more intense inflammatory reactions and more pronounced activity of eosinophils and mast cells in this NSAID hypersensitivity form of NP. Although few previous studies have addressed differences in HSP70 and ECP production between patients with and without aspirin hypersensitivity, we found no studies that addressed differences in tryptase, substance P and CC16 production between the two CRS phenotypes.^24^, ^25^ Kikuchi et al.^24^ analyzed HSP70 gene polymorphisms in Japanese patients with AERD and found the association between HSPA1B‐179C>T and HSPA1B1267A>G gene sequence variations and development of AERD. Stevens et al.^25^ found higher concentrations of ECP in tissue samples of patients with AERD than in patients without aspirin sensitivity, indicating the higher level of eosinophilic inflammation in the nasal/sinus mucosa of patients with hypersensitivity to NSAIDs.

HSP70 has numerous immunological functions, but its role in the pathogenesis of CRS has not been sufficiently investigated. In patients with inflammation of the nasal mucosa, the main source of HSP70 is the respiratory epithelim, but also activated eosinophils, mast cells and cells that have undergone the process of apoptosis.^3^, ^4^, ^5^ HSP70 stimulate the antigen‐presenting cells to produce several proinflammatory cytokines.^3^, ^4^, ^5^ In an immunohistochemical study, the authors demonstrated higher expression of HSP70 in eosinophilic form of NP than in noneosinophilic form.^26^ HSP70 activates the NF‐κB pathway of cytokine/chemokine production, resulting in the attraction of eosinophils to the site of inflammation and enhancement the survival of these cells in NP.^27^ This could be the explanation for our obtaining a positive correlation between HSP70 and ECP in both groups of patients with NP, with and with no aspirin sensitivity.

Eosinophils have a crucial role in damaging the nasal mucosa. Granules of activated eosinophils contain toxic ECP and histological investigations have shown cytotoxic effects of ECP on mucous membrane, resulting in edema of subepithelium and lysis of epithelium.^28^, ^29^ A previous investigation demonstrated that after the intranasal corticosteroid therapy, the levels of ECP in nasal fluid significantly decrease.^17^ So, ECP levels in nasal secretions can be used to assess the efficacy of treatment. The role of mast cells is very important in the pathogenesis of allergic form of chronic rhinitis. However, some investigations suggest its importance in the pathophysiological processes underlying CRSwNP.^18^, ^19^ Mast cells release proteolytic enzyme tryptase together with histamine during the process of degranulation. Due to a much longer plasma half‐life compared with histamine, tryptase is considered superior to histamine as a marker of mast cell function.^18^, ^19^ Our results also suggest a significant role of mast cells in the pathogenesis of NP, especially in AERD form. In the CRSwNP group, tryptase and ECP are found to be in positive correlation. It could be explained by the fact that after activation, mast cells release chemokines that attract eosinophils to the site of chronic inflammation.^18^, ^19^

The sensory nerves in the nasal mucosa contain several neuropeptides, but only substance P is well established. However, data concerning the neuropeptide secretion from NP tissue is poorly available. In the nasal mucosal layer, substance P stimulates vasodilatation, mucus secretion, as well as vascular permeability.^30^ Kühn and Arnold^31^ in their immunohistochemical investigation demonstrated that substance P has a similar distribution in noninflamed nasal mucosa and inflammatory polyps. The results obtained by this study, however, suggest a higher level of substance P production in patients with NP than in subjects without nasal inflammation, but we found no statistical difference between substance P levels in the CRSwNP and AERD patients. Our results also show a negative correlation between substance P and the LMS and ES in the CRSwNP patients. Nasal function is, among other, regulated by neurogenic mechanisms. Rich innervation of the nasal/paranasal sinus mucosa together with plenty of immunocompetent cells, seems to be functionally interrelated.^32^ This immunoneurological interaction plays a highly significant role in modulating an immunological response in patients with allergic rhinitis.^32^ However, it is possible that the release of substance P in the pathologically altered nasal mucosa of NP is not the same as in patients with allergic rhinitis. NP themselves are formations that are much less innervated in relation to the unaltered nasal mucosa.^1^, ^2^ In NP, mainly mucosa of the ethmoidal labyrinth and maxillary sinus underwent remodeling in the direction of NP formation. Accordingly, we might assume that the increased concentration of substance P in our NP patients is released from the remaining part of the nasal mucosa with no polypoid degeneration, which could perhaps explain the absence of positive correlation between the extent of disease and concentration of substance P in nasal secretions. However, in our results there is no evidence for this, so our assumption is suggestive and requires further investigation.

Our investigation demonstrates a negative correlation between CC16 and nasal symptoms in the AERD group, as well as a negative correlation between CC16 and tryptase in the CRSwNP and the control group. The main sources of CC16 are non‐ciliated Clara cells of the respiratory tract.^6^, ^17^ This is a strong anti‐inflammatory and antiallergic mediator which inhibits enzyme phospholipase A2 in the airways of patients with chronic nasal inflammation and in air pollutant‐exposed subjects.^6^, ^17^ Eosinophilic inflammation in patients with NP decreases the levels of CC16.^6^, ^17^ Also, the concentration of CC16 in the nasal fluid is inversely corelated with the level of ECP and eotaxin‐2 in patients with NP and perennial allergic rhinitis.^6^, ^17^, ^33^ A negative correlation between CC16 and tryptase in nasal secretions of the current study participants indicates a significant sensitivity of Clara cells to tryptase that also can damage the epithelium. Following topical corticosteroid use, the concentrations of CC16 increased in both perennial allergic rhinitis and NP patients, suggesting that CC16 could be a reliable marker for the assessment of the recovery function of sinonasal mucosa.^17^, ^33^ At first glance, it is difficult to explain the fact that concentration of CC16 in the control group is positively correlated with the intensity of nasal symptoms. However, CC16 concentration is negatively correlated with symptoms in patients with NP, where the nasal mucosa has undergone structural changes, but not with symptoms in subjects with healthy nasal mucosa where, in our control participants, nasal obstruction due to deformation of the nasal septum predominates.

Due to financial limitations, however, it was not possible for us to perform an immunohistochemical investigation to evaluate the potential of the nasal mucosa/NP tissue to produce these mediators. However, the aim of this research was also to show that the monitoring concentrations of biochemical parameters make it possible to better understand the complex inflammatory processes in NP. Determining the mediators of inflammation in nasal secretions could be useful as being a much simpler method for observing these processes in relation to immunohistochemistry.

## 5 CONCLUSIONS

The results obtained in our study clearly demonstrate higher ECP, HSP70, and tryptase and lower CC16 levels in nasal secretions of the patients with AERD, suggesting an increased production of mediators of eosinophil and mast cell function, and a decreased production of biomarker of respiratory epithelium function in the patients with aspirin sensitivity. Clinical and biochemical parameters of chronic inflammation correlate in different ways in the AERD and nonaspirin sensitized CRSwNP patients, suggesting the possibility that different inflammatory mechanisms are involved in the development of these two forms of the disease. Positive correlations in the concentrations of ECP and HSP70, as well as between ECP and tryptase and a negative correlation of CC16 and tryptase in nasal secretions suggest the presence of an intense and orchestrated activity of respiratory epithelium, eosinophils and mast cells in the pathogenesis of NP.

## AUTHOR CONTRIBUTIONS

*Concept and design*: Aleksandar Perić, Gordana Cvetković, Danilo Vojvodić. *Participant recruitment*: Aleksandar Perić, Dejan Gaćeša, Gordana Cvetković. *Experiment execution*: Aleksandar Perić, Dejan Gaćeša, Gordana Cvetković, Danilo Vojvodić. *Data analysis*: Aleksandar Perić, Gordana Cvetković, Danilo Vojvodić. *Drafting and critical revision of the manuscript*: Aleksandar Perić, Dejan Gaćeša, Gordana Cvetković, Danilo Vojvodić. All the authors contributed to the article and approved the submitted version.

## CONFLICT OF INTEREST STATEMENT

The authors declare no conflicts of interest.

## Data Availability

All data in the present study are available from the corresponding author upon reasonable request.
